# Modeling of Neurodegenerative Diseases: ‘Step by Step’ and ‘Network’ Organization of the Complexes of Model Systems

**DOI:** 10.3390/ijms24010604

**Published:** 2022-12-29

**Authors:** Viacheslav Igorevich Pasko, Aleksandra Sergeevna Churkina, Anton Sergeevich Shakhov, Anatoly Alexeevich Kotlobay, Irina Borisovna Alieva

**Affiliations:** 1 Biological Faculty, Lomonosov Moscow State University, 1–12, Leninskye Gory, 119992 Moscow, Russia; 2 Faculty of Bioengineering and Bioinformatics, Lomonosov Moscow State University, 1–73, Leninskye Gory, 119992 Moscow, Russia; 3 A.N. Belozersky Institute of Physico-Chemical Biology, Lomonosov Moscow State University, 1–40, Leninskye Gory, 119992 Moscow, Russia; 4 Lopukhin Federal Research and Clinical Center of Physical-Chemical Medicine of Federal Medical Biological Agency, 1a Malaya Pirogovskaya St., 119435 Moscow, Russia

**Keywords:** neurodegenerative diseases, model organisms, disease modeling, proteinopathies, organoid models

## Abstract

Neurodegenerative diseases have acquired the status of one of the leading causes of death in developed countries, which requires creating new model systems capable of accurately reproducing the mechanisms underlying these pathologies. Here we analyzed modern model systems and their contribution to the solution of unexplored manifestations of neuropathological processes. Each model has unique properties that make it the optimal tool for modeling certain aspects of neurodegenerative disorders. We concluded that to optimize research, it is necessary to combine models into complexes that include organisms and artificial systems of different organizational levels. Such complexes can be organized in two ways. The first method can be described as “step by step”, where each model for studying a certain characteristic is a separate step that allows using the information obtained in the modeling process for the gradual study of increasingly complex processes in subsequent models. The second way is a ‘network’ approach. Studies are carried out with several types of models simultaneously, and experiments with each specific type are adjusted in conformity with the data obtained from other models. In our opinion, the ‘network‘ approach to combining individual model systems seems more promising for fundamental biology as well as diagnostics and therapy.

## 1. Introduction

Neurodegenerative diseases, once rare in developed countries, are becoming a major public health threat and a leading cause of death in parallel with an increase in life expectancy. These diseases are quite diverse in their pathophysiology—with some causing memory and cognitive impairments and others affecting a person’s ability to move, speak and even, breathe. Effective methods of treating neurodegenerative diseases are extremely necessary, but modern medicine still does not have such methods. Targeted treatment methods can appear only after an in-depth study of the causes and mechanisms of each disease. All diseases of this type have several common features, such as prolonged development (years or decades), mass death of neurons and neuroglia, and the formation and spread of protein aggregates with an abnormal tertiary structure. Among the main reasons for their development are:

1. Aging and the loss of ability to maintain normal processes of cell death and the utilization of abnormal proteins associated with aging. The most characteristic cases are taupathies (Alzheimer’s disease) and synucleinopathy (Parkinson’s disease);
2. Mutations, most often expansion of trinucleotide repeats (Huntington’s disease; spinocerebellar ataxia types 1, 2, 3, 6, 7 and 17);
3. Inflammatory processes in the nervous system;
4. The effect of adverse environmental factors.

Affecting mainly the elderly, neurodegenerative diseases increase the number of disabled people, which creates an additional burden on families, the economy and social services. In addition, in recent years cases of neurodegenerative diseases are becoming increasingly frequent in younger people; the development of Huntington’s disease was reported in 9-year-old patients [1,2]. Moreover, there is growing evidence of a link between neurodegenerative and psychiatric diseases, often based on the same mechanisms [3].

Neurodegenerative diseases can have both a hereditary nature or develop sporadically. The development of the neurodegenerative process is based on disorders in the structure and function of certain proteins. Abnormal aggregation and the toxic effect of these proteins on cells lead to the occurrence of diseases. Neurodegenerative pathologies include tauopathies (Parkinson’s disease), amyloidosis (Alzheimer’s disease), α-synucleinopathies (Levi’s bodies in Parkinson’s disease), TDP-43 (TAR DNA-binding protein 43) and SOD1 (superoxide dismutase 1) proteinopathy (amyotrophic lateral sclerosis), prionopathy and others. A separate group consists of polyglutamine diseases resulting from a congenital mutation—triplet expansion of CAG (polyglutamine—polyQ) in certain genes. Huntington’s disease is an example of such a neurodegenerative disease [4].

The study of the neurodegenerative disease models and the modification and rethinking of old models along with the creation of fundamentally new systems are important for several reasons. The first reason is that our knowledge of neurodegenerative diseases is expanding rapidly, and old models are no longer adequate. The second reason is that drugs and treatments that were projected to be effective during development and testing in cell cultures have shown their failure in clinical trials. These facts indicate the insufficiency of the existing model systems [5]. Finally, the most important reason is that new models will allow us to combine data obtained from in vitro tests, clinical trials and studies on animal models, as well as involve approaches from related or non-biological fields, including bioinformatics. One of the main problems in modeling neurodegenerative diseases is the lack of a universal model for all the mechanisms and processes involved in neurodegeneration. Therefore, it is advisable to analyze the main advantages and limitations of models and propose a synthetic approach to their use as part of an integrated complex of model systems.

## 2. Basic Requirements for Models of Neurodegenerative Disorders

One way to learn about how a disease works to destroy the normal functions of an organism is to develop a model system that recapitulates the hallmark characteristics of the disease.

The main requirement for a neurodegenerative disease model is the ability to reproduce characteristic pathological processes resulting from specific molecular and cellular disorders. The importance of this requirement is due to the fact that most modern methods of treatment do not address the basic mechanisms of neurodegenerative diseases, but only relieve symptoms [6,7]. In addition to reproducing the basic mechanisms, the model must also undergo appropriate functional and morphological changes at the cellular, tissue and organism levels.

### 2.1. Variability and Phenotypic Plasticity

Numerous studies have shown that neurodegenerative diseases can have a variety of mechanisms of occurrence [8,9,10,11]. The same symptoms may be due to different causes. Conversely, the same factor under certain conditions can cause several neurodegenerative diseases with different symptoms [8,12,13]. The development of a particular symptom, or the disease as a whole, is due to the interaction of many factors, both internal and external. Even if the body has disorders on the molecular or cellular level that can lead to the development of a neurodegenerative disorder, they may not manifest themselves if the body is in favorable conditions [14]. Conversely, unfavorable conditions lead to the manifestation of disorders, the occurrence of which was previously successfully suppressed by the body [12]. The key property of neurodegenerative disease models should be the ability to change under the influence of negative environmental factors, as well as to change the course of normal life processes in general. Only in this way will the models fully simulate neurodegenerative processes.

### 2.2. Biochemical Correspondence of the Model to the Organism under Study

As has already been mentioned, to obtain the most accurate results, a model of a neurodegenerative disease should approximate the ‘original’ organism from the point of view of metabolic disorders as well. The model should not only reproduce the symptoms of the disease but also copy the pathological process at the molecular level and the signaling cascade (or proteinopathy) that is disturbed in a specific disease. It has been proved that models of hereditary forms of neurodegenerative diseases, even in the case of incomplete compliance with phenocopic changes occurring under the influence of unfavorable environmental factors, make it possible to fully reproduce the molecular mechanisms underlying the disease [15]. This approach allowed the rejection of ineffective and dangerous methods of treatment [16]. For the most complete conformity to the human body, a number of pathological processes corresponding to the disease should occur in the animal model at many levels: from molecular-genetic to the general organism, including biochemical processes and metabolism. Special attention in the models should be paid to the problems of general aging of the body and the consistent development of symptoms caused by the gradual accumulation of mutant or damaged proteins. The model should assume the gradual development of the symptoms of the corresponding disease since this is critical in terms of studying the development of the disease and potential treatment. Mutations in model organisms should not cause a rapid disruption of nervous activity or a general disruption of metabolic processes. The model should respond selectively to genome disturbances and the spread of damaged proteins throughout the body, accurately copying the symptoms of the ‘original’ disease.

### 2.3. The Rate of Reproduction and Model Response to Impact

The approach to the treatment of any neurodegenerative disease involves three stages: diagnosis, actual treatment and further evaluation of its effectiveness. Each of these stages is important and can become an object of modeling. The time frame is no less important since the development of neurodegenerative pathologies can last for years and even decades. Thus, the development of Alzheimer’s disease takes an average of three years and Creutzfeldt-Jakob disease—within nine to ten years [17,18]. The disease may have a very long incubation period or be the result of many years of exposure to negative environmental factors such as heavy metal pollution [12]. Models of neurodegenerative diseases should, as far as possible, reproduce the corresponding time intervals between key events accompanying the development of neuropathology, without sacrificing the quality and accuracy of modeling. In addition, for animal models (especially those that replicate pathologies caused by inherited genetic defects) the rate of reproduction, generational change, and the lifespan of an individual is of importance.

### 2.4. Functional Correspondence of the Model to the Organism under Study

This requirement applies to animal models of neurodegenerative diseases, more precisely, to models based on vertebrates, since only they have a sufficiently developed nervous system and complex types of behavior comparable to the higher nervous activity of man.

The described complex types of behavior are associated with the fact that the nervous system is never in a stable state, as it is constantly rebuilt and modified. The formation of reflex arcs and the memorization of information and learning require the restructuring of neural connections, the decay of old ones and the formation of new ones. Moreover, the nervous system is rebuilt in the process of body growth and maturation. One should not forget that the nervous system is not protected from damage, and inflammation and regeneration processes take place in it. The processes of neuroplasticity are especially strongly activated after craniocerebral injuries and stroke. In general, neuroplasticity processes can be divided into two types: regeneration (the formation of new cells or new synaptic contacts) and functional restructuring (occurs without changing the number of cells) [19]. These processes should also be taken into account by researchers when creating model systems.

## 3. Prokaryotic Models

### 3.1. General Characteristics of Prokaryotic Models

Prokaryotic organisms are routinely used in modern biology and biotechnology to produce substances with set properties (mainly proteins), including mutant proteins characteristic of certain neuropathology (act as protein factories). Thus, researchers have the opportunity to study the proteins involved in neurodegeneration without using human material to identify their structure, aggregation mechanisms, etc.

Today prokaryotes are increasingly used to create models of the ‘microbiome-brain-gut’ system in neurodegeneration studies [20]. These models are not pure prokaryotic, since the model includes a vertebrate as a host. It is shown that malfunction of the blood-brain barrier [21] can lead to the entry of bacterial amyloids into the bloodstream, into the brain and further prion-like dissemination of the abnormal protein structure in the nervous tissue. In addition, the process can begin with enteroendocrine and M-cells in the intestinal epithelium and be transmitted to the brain through the autonomic nervous system via a bidirectional pathway [22]. Conversely, pathogen-associated molecular patterns (PAMPs) can lead to the overactivation of the immune system, autoimmune reactions, and inflammation in the nervous system [22,23,24,25]. The interest of neuroscientists in the models of the “microbiome-brain-intestine” system is growing every day since the technologies for creating bacterial factories producing mutant proteins are well developed and are easy enough to introduce into the host body.

### 3.2. The Role of the Intestine’s Microflora in the Development of Neurodegenerative Diseases: The ‘Microbiome-Brain-Gut’ Axis

The gut microbiome, together with the immune, endocrine and all three sections of the autonomic nervous system (parasympathetic, sympathetic and enteric) forms a complex that allows bacteria to influence the central nervous system [20]. The metabolic products of bacteria interact directly with the central nervous system. As a result, the development and functioning of the brain in both normal and pathological conditions is largely determined by the mutual transmission of signals between the brain and the intestine [26].

In earlier works, the main research tool in this area was model animals with impaired microflora. Now models of the intestinal microbiome in vitro allow us to more accurately analyze the composition of the microflora of the gastrointestinal tract.

Several in vitro microbiome systems have been described, among which three main types can be distinguished:

1. SHIME—The Simulator of the Human Intestinal Microbial Ecosystem. The system is a complex consisting of bioreactors connected in series with peristaltic pumps. In each bioreactor conditions specific to a particular section of the digestive system are created (an anaerobic environment and optimal temperature are maintained in all bioreactors). The reactors correspond to the following sections of the gastrointestinal tract: stomach, small intestine, ascending colon, transverse colon, and descending colon [27];
2. SIMGI—SIMulator of GastroIntestinal tract. This system is similar to SHIME in many ways, but there are some differences. This model is not only fully automated but also controlled by a computer. Moreover, the gastric compartment has a peristaltic mixing system [26];
3. Organ-On-A-Chip Systems. These include microfluidic systems, which are ultra-compact (within fractions of a millimeter) bioreactors containing organotypic cell cultures capable of reproducing the pathophysiological processes in miniature. In the case of modeling the ‘microbiome-brain-gut’ axis, the role of these systems is to reproduce the processes of the transfer of bacterial neurotoxins through various microanatomical barriers, including the blood-brain barrier [28];

### 3.3. Transgenic Bacterial Models

The main sphere of application of transgenic bacterial models of neurodegenerative diseases is the synthesis of pathological proteins and the subsequent study of how they fold, interact and aggregate dynamically [29].

*E. coli*, being one of the most thoroughly studied prokaryotic organisms, turned out to be not the most convenient model organism for use as a ‘protein factory’. The reasons are the need for the constant application of antibiotics to maintain the culture, low transformation efficiency associated with the loss of plasmids, as well as problems at the stage of formation of the final product such as the violation of the formation of secondary and tertiary structures, protein aggregation and the absence of the necessary post-translational modifications. Therefore, the search for optimal cell factories should be carried out among bacterial species adapted to more diverse and extreme conditions [29].

Prions are a key factor that brings together a variety of disorders of the nervous system, from Creutzfeldt-Jakob disease to amyotrophic lateral sclerosis. They are a group of a wide variety of protein molecules that have an abnormal tertiary structure that can transfer their structure to homologous proteins, as well as form insoluble aggregates that gradually accumulate and are the main cause of the detrimental effect of prions on the nervous system [30]. Such a sequential process of accumulation causes a long duration of neurodegenerative process development.

Bacterial cells can be used as ‘protein factories’ that synthesize prions specific to humans or other mammalian organisms. For instance, a recombinant mouse prion protein (recMoPrP) produced in transgenic E. coli has been used to study polymerization and amyloid fibril formation. Further, fibrils consisting of recMoPrP (89–230) were administered intracerebral to experimental mice. The mice developed neurological dysfunction on the 380th to 660th day after the administration [31]. This study once again proved the ability of prion proteins to replicate at a high rate and participate in the development of neurological disorders and demonstrated, in this particular case, the effectiveness of using bacterial ‘protein factories’.

## 4. Yeast Models

### 4.1. The Role of Yeast Models in the Study of Neurodegenerative Diseases

The value of the yeast *Saccharomyces cerevisiae* as a model organism lies in the fact that many of the biochemical and signaling pathways present in the human cell are highly conserved and perform similar functions in yeast cells. Evolutionary conservatism underlies the main method of working with yeast models. After a gene is found in the human genome that can be associated with a particular pathology, a line of yeast is created, in the genome of which there are mutations in a homologous sequence. If the homologous gene is absent in *S. cerevisiae*, then the corresponding defective gene is introduced into the genome of the model. In both cases, if the defective gene is responsible for the development of pathologies (in particular, neurodegenerative disorders), then the corresponding phenotype develops in yeast cells [32].

Yeast models, due to their simplicity, are not suitable for reproducing the complex symptoms of neuropathology such as behavioral disorders or changes in the structure of brain tissue. Therefore, the main symptoms modeled in yeast are the accumulation of abnormal proteins, as well as the disruption of the normal course of cell death and autophagy.

The functioning of all intracellular mechanisms aimed at combating proteins with an abnormal tertiary structure is ensured by the functioning of the chaperone and ubiquitin-proteasome systems of the cell [33]. Thus, yeast models serve primarily to study the mechanisms of neurodegenerative-associated protein accumulation and the functioning of the protein degradation systems (chaperones, proteasomes and autophagy).

### 4.2. Modeling of Disorders in Chaperone Functioning

**Figure 1:**
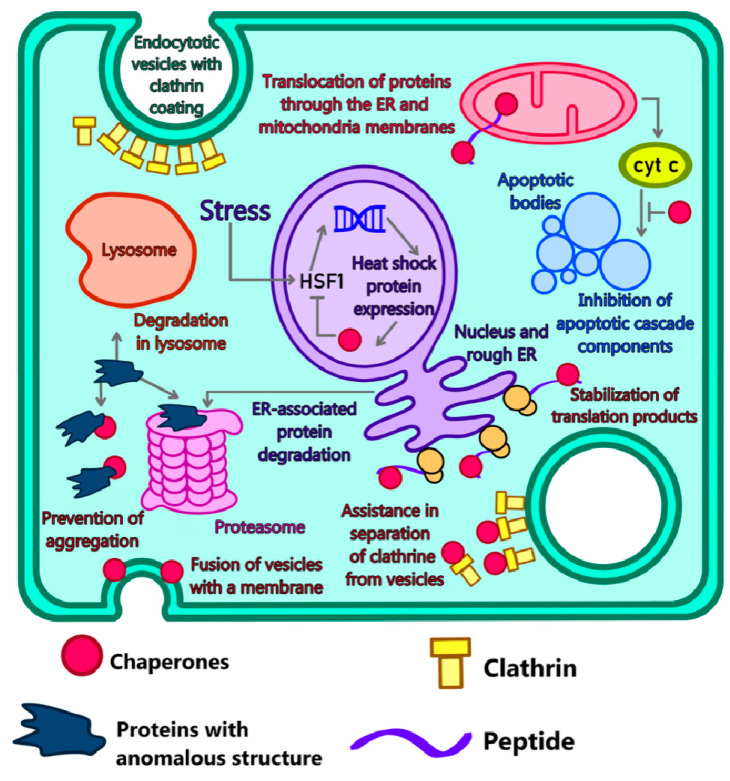
Diagram of intracellular chaperone system to fight abnormal proteins. Acting as a kind of assistant for the formation of a normal tertiary protein structure or, conversely, its degradation, chaperones are a universal system for preventing proteinopathies, which are the main cause of neurodegenerative diseases.

Molecular chaperones can be called the first line of defense against neurodegenerative diseases. They eliminate misfolded proteins, preventing their aggregation and the formation of amyloid structures [33,34,35]. Although the information necessary for peptide folding and formation of the correct tertiary structure is contained in the amino acid sequence, the probability of misfolding is quite high since there are many other molecules and molecular complexes inside the cell that can affect the correct folding. In addition to that, the protein may be initially predisposed to misfolding due to the presence of mutations that lead to errors in the amino acid sequence of the polypeptide chain, which causes the occurrence of hereditary forms of neurodegenerative disorders [36,37,38]. To protect against such errors during evolution, a specific chaperone system arose, aimed at combating misfolded proteins in all parts of the cell [32] (Figure 1). Normally, chaperones assist in the formation of functional tertiary structures of peptide chains after their synthesis on ribosomes. In addition, chaperones support the translocation of proteins through membranes of the endoplasmic reticulum and mitochondria. The role of chaperones in protein degradation consists of directing ubiquitinated proteins both from the endoplasmic reticulum and cytoplasm into proteasome-mediated degradation and mediating lysosomal degradation. Besides that, chaperones play key roles in system cellular processes, such as regulation of transcription factors during the heat stress response, clathrin coating dismantling, and the fusion of vesicles with cell membranes in exocytosis, and apoptosis. One of the main dangers associated with abnormal proteins is their ability to spread, affecting healthy proteins and changing their structure. In other words, they behave like typical prions. The studies carried out on yeast have not only helped to understand the nature of the prion distribution as such, but also the role of chaperones in their distribution. Although chaperones are supposed to rid the cell of misfolded proteins in the case of β-amyloid, they, on the contrary, contribute to their distribution. Experiments on yeast models have shown that chaperones fragment growing β-amyloid fibrils, which leads to an increase in the number of fibril growth centers and their further distribution [39].

### 4.3. The role of Proteasomes and Their Interaction with Abnormal Proteins

Proteasomes cleave misfolded and mutated proteins into short peptide fragments. The active 26S proteasome is formed from a 20S subunit (core) and several 19S (regulatory) subunits. Experiments with yeast models have shown that the 20S subunit is inhibited by three-dimensional structures resulting from the oligomerization of proteins that are specific for neurodegenerative diseases such as Alzheimer’s (β-amyloid), Parkinson’s (α-synuclein) and Huntington’s disease (HTT protein) [40].

### 4.4. Autophagy

Autophagy is an intracellular process necessary to maintain cell homeostasis. During autophagy, unnecessary and damaged cellular organelles and proteins are utilized. Autophagy is a critical process in a multicellular organism during embryogenesis, and in aging, and also plays a role in the development of diseases, including heart failure [41], myopathies [42] and neurodegenerative diseases [43]. Autophagy is also present in unicellular organisms, where it primarily serves as a reserve source of nutrients in the case of stress. Moreover, the main mechanism of autophagy was initially identified in yeast. A typical object of study of autophagic processes in a unicellular organism is *S. cerevisiae* [44]. Yeast models have been used to elucidate the exact molecular mechanisms of autophagy and key players in the signaling cascades associated with it, such as the Atg family of proteins. Autophagic mechanisms are highly conserved (including autophagic pathways involving TOR and PKA) and have been found to be present in both yeast and humans, with great similarity [45]. Studies on mammalian cells have shown the effect of hormonal regulation, energy status and nutrient levels on autophagy. In addition, environmental stress factors such as hypoxia, heat stress and the accumulation of reactive oxygen species are important (discussed in [46]).

Autophagy in yeast is known in sufficient detail at the molecular level. In addition, the autophagic process in *S. cerevisiae* proceeds synchronously in a homogeneous culture and depends on the amount of nutrients in the medium, which makes it easy to control it in experiments [47]. Thanks to all of the above and the abundance of developed methodological approaches, yeast is a convenient model for studying autophagy. However, the autophagy impact on the multicellular organism development and functioning, especially the nervous system, cannot be completely estimated only by yeast models.

## 5. Models Based on Invertebrates

### 5.1. Advantages of Multicellular Models

Models of neurodegenerative diseases based on multicellular animals have several specific properties that are not available for unicellular systems. The most significant is the ability to simulate the systemic processes of the human body: the functioning of intercellular signaling pathways, the restoration of the nervous system after cell death and the distribution of proteins with an abnormal tertiary structure inside the body [15,48,49].

One of the most important fields of application for animal models of neurodegenerative diseases is aging. Aging is a universal process in which the normal functions of cells in multicellular organisms are disrupted; there is also a loss of the tissues’ ability to renew and maintain a functional state. Aging is characterized by a number of pathological processes caused by:

1. The loss of the ability of cells and tissues to maintain normal cell death processes;
2. Disorders in the utilization of misfolded and aggregated proteins that are toxic;
3. The accumulation of cellular debris due to disorders of the autophagolysosomal apparatus;
4. The uncontrolled course of inflammatory processes, mainly in the CNS.

The aggregation of abnormal proteins is considered one of the main causes of the development of neurodegenerative diseases associated with aging. The main questions which are yet to be answered by researchers of each specific disease are as follows:

1. What does the toxic activity of abnormal protein aggregates look like at the molecular level?
2. Is there a relationship between aggregation and toxicity?
3. Do protein aggregates observed in neurodegenerative diseases have any common properties, including pathological ones?
4. Why are these diseases associated with aging?
5. What causes the development of pathologies associated with a particular cell type [50]?

### 5.2. Invertebrates as Models for Neurodegenerative Disorders

The role of multicellular models is to approximate molecular biology data from single-cell models to fundamental processes such as growth, morphogenesis, aging, stress adaptation, synaptic network formation, and neurodegenerative processes. Models of neurodegenerative diseases based on invertebrates have several characteristic features, which could provide answers to the above questions, the most significant of which are the following:

(A) Presence of specialized cells and tissues. Unlike previous types of models, in a multicellular organism, several types of cells that perform different functions are combined into a system where they have a direct or indirect effect on each other. An example is the interaction of neuroglia with neurons, which is one of the key aspects of the accumulation and distribution of amyloids in the nervous system [51].

(B) Evolutionary relationship. Despite the fact that the external differences between humans and Drosophila seem colossal, at the molecular level there are significant similarities and conservatism, especially in the mechanisms of morphogenesis, autophagy, and cell death. An example is the signaling cascades including Wnt proteins. Wnt proteins are a family of secreted glycoproteins responsible for patterning embryonic development, gastrulation, formation of synaptic connections, axonal remodeling, regulation of cell proliferation, stem cell development, and many other processes. Wnt protein binds to the frizzled receptor (Fz), which has seven transmembrane domains and is topologically similar to G-protein coupled receptors, which leads to the separation of the GSK3, Axin, and APC protein complexes. In turn, this prevents the phosphorylation of β-catenin. This protein, in its unphosphorylated form, enters the nucleus and binds there to transcription factors [52].

An example of a direct application of evolutionary conservatism in the field of neurodegenerative disease models is the neuroprotective effect of lithium ions. From ancient times to the present day they have been used to treat nervous disorders [53,54,55]. Research aimed at studying the mechanism of action of lithium ions on *Dr. melanogaster* has shown that these ions affect the canonical (β-catenin-dependent) Wnt pathway [56]. Transgenic flies capable of synthesizing and accumulating β-amyloid were used in the study of Jans et al. [55]. During the development of Alzheimer’s disease, Wnt signaling is disrupted because migrating β-amyloids increase the level of GSK3 in the intercellular space, which, in turn, leads to the inactivation of β-catenin and inhibition of the expression of proneural genes. Proneural genes are known for their ability to bring brain stem cells out of dormancy, stimulating their proliferation and differentiation [57,58,59]. Since the loss of the brain’s ability to renew itself and recover from damaging effects is one of the key factors in the development of neurodegenerative disorders, the use of lithium preparations that inhibit GSK3 will have a complex effect on the central nervous system. A thorough study of such mechanisms (which change rarely in the course of evolution) and molecular homologies would not only explain the action of existing drugs but also create a foundation for the development of new, more effective drugs.

(C) The presence of nervous activity and behavior. Behavioral changes, especially in basic responses such as foraging and spatial orientation, are among the most easily recorded signs. Since the main goal of research in neurodegenerative diseases is the restoration of normal intellectual and social functions in patients, behavioral changes in animal models are still of great value to researchers, because due to the relative evolutionary conservatism of many molecular and signaling pathways, they allow for the establishment of parallels between the behavior of experimental animals, including invertebrates, and humans suffering from neurodegeneration [60].

(D) Autophagy. Invertebrates are also a classic model for studying autophagy processes in neurodegeneration and are often used to study several signaling pathways and molecular cascades.

### 5.3. Use of C. elegans as a Model for Studying the Processes of Cell Death and Autophagy

**Figure 2:**
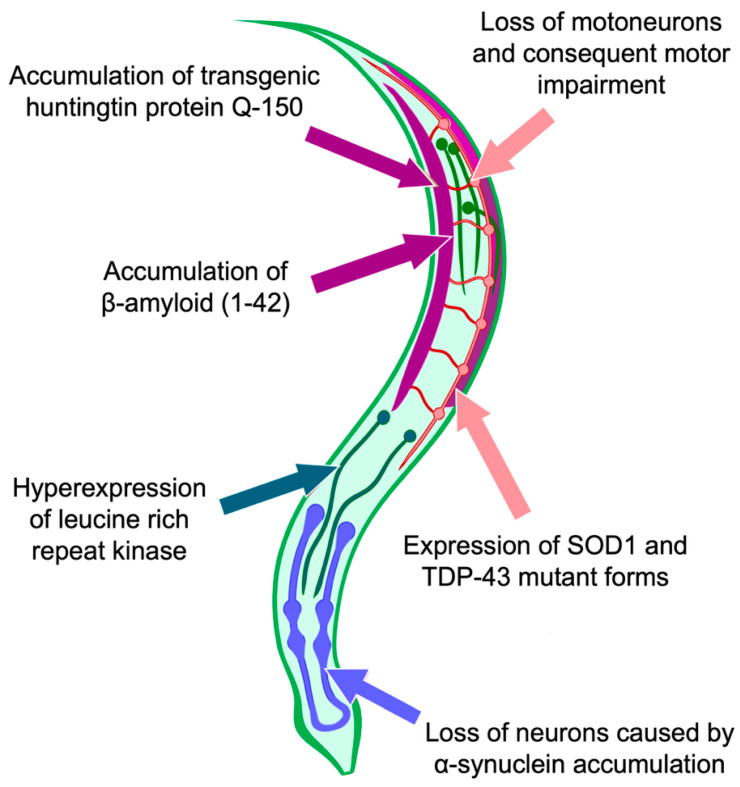
The study of complex action of factors leading to the loss of neurons and the connectome disruption integrity in the *C. elegans* body. The presence of neurons that differ in functions and types of neurotransmitters used, together with a strictly determined number of cells, make *C. elegans* an optimal object for connectome studies. Expression of mutant forms of superoxide dismutase and TAR DNA binding protein 43 results in motor neuron loss in *C. elegans*. This model serves to showcase the impact of processes, which are considered to be key mechanisms of amyotrophic lateral sclerosis development, Accumulation of huntingtin (number of CAG repeats 150) and amyloid β (the number of residues involved in the beta sheets formation is 42) demonstrates devastating consequences of Huntington’s disease and Alzheimer’s disease, respectively. Disrupted connectome in the case of Parkinson’s disease can be modeled through hyperexpression of leucine-rich repeat kinase. Disrupted connectome in the case of Parkinson’s disease can be modeled through hyperexpression of leucine-rich repeat kinase.

The nematode *Caenorhabditis elegans* is a quite convenient model for the study of cell death. The advantage of this model is the constancy of the cellular composition. Adult hermaphroditic individuals have 959 somatic nuclei, and the number of nerve cells is 302 [61]. All the connections formed by nerve cells are also fixed [62]. This allows describing of a connectome, comprising a complete structure of the nerve connections in the body The connectome makes it possible to comprehensively describe the structure and functional role of synaptic connections both within the brain and within the whole body, including neurodegenerative processes (Figure 2). Connectomics-based approaches allow the nervous system to be modeled as a network of interconnected structures, which makes it possible to draw conclusions about how dynamic changes in the function of the nervous system can be associated with structural changes.

The advantage of using *C. elegans* as a connectome model lies not only in the small number of cells and its determinism but also in the permeability of the worm body to light, which makes it possible to conduct studies with fluorescent proteins included in nerve cells [63]. For example, the complex action of factors leading to neuronal death, such as mutations in superoxide dismutase-1 and in the TDP-43 protein, leads to the appearance of the areas in the worm body where neurons and glial cells die; therefore, when the body of a worm is studied with the help of a fluorescent microscope, these areas look dark [64,65]. Approaches based on connectomics, in combination, with microscopic methods, allow us to clearly map the areas of pathological disorders that occur in specific parts of the nematode nervous system. This makes *C. elegans* a popular model for studying various neurodegenerative diseases.

## 6. Mammals-Based Models

### 6.1. Advantages of Using Mammals

The use of mammals as model organisms is due to their physiological, anatomical and genetic similarity to humans. For centuries mammals have acted as a ‘visual aid’, a source of knowledge about the structure of the human body—the data obtained during the autopsy of animal corpses have served to create an increasingly detailed diagram of the human body structure since ancient times. Moreover, although even then the researchers realized that human and animal organisms are not absolutely identical, the fact that there is a significant similarity between humans and other mammalian species was undeniable.

Today animal models, including neurodegenerative diseases, while still playing an important role in the study of human diseases, are experiencing serious competition from alternative systems, especially in vitro culture models. The reason is the difficulty of reproducing the symptoms of neurodegenerative disorders in the animal body. One of the main approaches in the degenerations study is the search for a new gene (or a mutant version of an already studied gene) responsible for the development of pathology, as well as proteins and protein complexes that can become disease markers. The data obtained form the basis of a model organism and act as a kind of guideline in its creation. The problem is that neurodegenerative processes in many cases occur sporadically without affecting the hereditary apparatus of the cell. In Alzheimer’s disease more than 90% of cases occur sporadically, and the exact cause of their development is unknown (it is assumed that in this case the main role is played by epigenetic mechanisms) [66]. In addition, there are significant genetic and biochemical differences between the organisms of model mammals and humans despite the evolutionary closeness, due to which the animal model is almost never a complete copy of the sick person organism in terms of phenotype [15]. However, their complete replacement remains impossible for several reasons:

1. The necessity to study the molecular mechanisms and mechanisms of initiation of proteinopathy. The neurodegenerative process can be considered in its entirety, considering the influence of all organs and systems of the body, even if phenotypically the degeneration is not fully manifested;
2. The relative ease of maintaining the model system;
3. The presence of age-related changes as a natural process inherent in the body of an animal. Aging, being a systemic process of reducing the body’s ability to maintain its functional state, is the main risk factor for a number of diseases, including neurodegenerative ones;
4. Highly organized immune system;
5. Presence of complex neural activity and behavior (especially in primate-based models).

### 6.2. The Use of Mammals to Model Aging Processes

For gerontological research, various animal models of mammals have been used for a long time and have shown success [67,68].

### 6.3. The study of Oxidative Stress Processes as a Factor in the Development of Neurodegenerative Diseases

Chronic oxidative stress is associated with damage to mitochondria and it is one of the causes of neurodegenerative disorder development [69]. Evidence from multiple studies suggests that mitochondria play a central role in aging-associated neurodegenerative diseases [8]. Mitochondria are able to influence the processes of cell death for many reasons, among which two main problems can be distinguished: the first one is mutations in mitochondrial DNA and oxidative stress, and the second—is the interaction of proteins specific for disorders of the nervous system with mitochondria [8].

The role of mitochondria and mitochondrial proteins in the development of neurodegenerative diseases is best studied for Parkinson’s disease. In most cases this disease is idiopathic, but there are rare hereditary forms, some of which are monogenic [70,71]. Observations have shown that the development of hereditary Parkinson’s disease may be due to a mutation in the genes of one of the five proteins responsible for the functioning of mitochondria—α-synuclein, parkin, DJ-1, PTEN-induced kinase 1 (PINK1), and leucine-rich repeat kinase 2 (LRRK2). It is believed that mitochondrial dysfunctions are the direct and one of the main causes of the death of dopaminergic neurons in Parkinson’s disease [48].

Mice have proved to be convenient animal models for the investigation of mitochondria and oxidative stress involvement in neurodegenerative processes due to the high degree of similarity with humans at the anatomical and molecular levels. At 25 weeks of age, test mice show symptoms of neurodegenerative disorders that simply could not be modeled in *Dr. melanogaster* or *C. elegans*: alopecia, cardiomyopathy, osteoporosis, anemia, sarcopenia and kyphosis [8].

### 6.4. The Use of Mammals to Study the Role of the Immune System in the Neuropathology Development

Autoimmune reactions are the risk factors for the development of neurodegenerative processes. Adaptive immune response cells, especially Th cells, play an important role in the pathogenesis of multiple sclerosis [72]. Experimental autoimmune encephalomyelitis has been the main animal model of multiple sclerosis for decades. It consists of inducing autoimmunity in mice after injection of peptides from myelin emulsified in an adjuvant [73]. Researchers have shown that myelin-reactive Th1 or Th17 cells can cause adaptive encephalomyelitis in mice; however, histopathological results obtained for CD4+ T cell populations differ from each other. Animals treated with Th1 cells had a more pronounced macrophage response, while mice with Th17 cells showed higher neutrophil infiltration [9]. These differences are significant because, although neutrophils and macrophages are phagocytic cells, only macrophages are antigen-presenting cells. They can then activate adaptive immune cells, which promotes a chronic inflammatory response. Moreover, the previously described data suggest that Th1 and Th17 cells play an important role in the development of lesions in MS, given that both effector populations can cause inflammation in the CNS and demyelinating lesions. However, their effector mechanisms differ from each other. Applications of existing and the creation of new experimental animal models are of significant importance for the investigation of neurodegenerative diseases due to the complicated structure of the immune system and its invaluable role in the progression of nerve cell death.

### 6.5. Behavioral Disorders Modeling

The ability to study complex behavioral reactions resulting from disorders of the structure and functions of the nervous system is a unique feature of animal models of neurodegenerative disorders. Experiments on mice have shown that an increased level of pro-inflammatory cytokines corresponds to a state similar to depression [74]. Mutations in the TDP-43 protein also affect behavior. TDP-43 is an important protein that affects the processing of a large number of target RNAs [75] and its ubiquitous deletion in mice results in embryonic death [76,77]. Though it is important to note that the spectrum of TDP-43-associated RNAs differs between species, and the changes in RNA processing caused by loss of function in TDP-43 differ significantly between mice and humans [15]. Mutations in TDP-43 lead to the development of some neurodegenerative diseases, for instance, amyotrophic lateral sclerosis. There are many models expressing human TLR-43 based on transgenic rodents. Overexpression of TDP-43 induces a lethal phenotype in mice regardless of the presence of the mutation. However, mice with an expression level close to endogenous TDP-43 develop mutant and age-dependent neurological disorders, including motor and cognitive disorders, motor neuron degeneration and neuromuscular disorders, with the exception of paralysis, which is not observed. TDP-43Q331K knockout animals develop mild cognitive dysfunction without spinal motor neuron degeneration [78]. Due to the complexity and specificity, modeling of behavioral reactions cannot be reproduced in any other way except using animal models of neurodegenerative diseases. We can expect the appearance of such new models on mice in the near future.

## 7. Organoid Models of Neurodegenerative Diseases

### 7.1. General Characteristics of Organoid Models

Organoids are called cultural model systems derived from stem cells and have a spatial structure that ensures their correspondence with human organs. In other words, the goal of creating organoid models is to reproduce a small copy of an organ under controlled in vitro conditions.

Approaches based on the use of animal models have made it possible to reveal the basic principles of the development of many pathologies. However, the results obtained when working with such model systems could not be directly extrapolated to the human body. There are a number of mechanisms that are specific to the human body that cannot be modeled in other animals. The most striking example is brain development. The human brain is a structure unique in its complexity, variability and functional plasticity. The processes of memory formation, conditioned reflexes and, ultimately, higher nervous activity, which underlies the evolutionary success of man as a biological species, are provided by constant changes in the structure of the brain, restructuring of synaptic connections, removal of old and the creation of new nerve circuits. At the same time, many mechanisms of the functional reorganization of the brain (especially the regions of the cerebral cortex responsible for rational activity) are unique to humans and cannot be modeled even in anthropoid primates. Differences already exist at the level of gene expression patterns. Thus, researchers from the Cold Spring Harbor Laboratory [79] analyzed gene expression patterns in different regions of the chimpanzee and human brain. It was shown that these patterns differ, and in one of the regions, these differences reach 10%. The greatest interspecies differences in expression were observed for duplicated genes. Another difference, even more significant from the point of view of the development of neuropathology and disorders of the brain structure, is structural differences. Significant variations in the density and number of neurons have been observed between different primate species, including between humans and chimpanzees [80]. Creating a model capable of reproducing the spatial structure of the brain would overcome these limitations. This is especially important for research in the field of traumatology since brain injuries can be one of the risk factors for the development of neurodegenerative diseases.

All types of models that have been discussed so far do not include human cells and tissues; therefore, they do not allow the direct ‘transfer’ of the obtained data to the human body. Even the smallest genetic and molecular differences could affect the signaling cascade leading to the accumulation of toxic proteins or, conversely, reduce the effectiveness of the drug that suppresses this process.

Thus, the emergence of three-dimensional cultures of human cells, based on stem cells obtained from various organs, has become an important step in overcoming these limitations.

### 7.2. Use of Organoid Models for Research in the Field of Regeneration and Traumatology

Organoid models open prospects not only for creating systems for searching for the neurodegenerative processes mechanisms and testing drugs, but also for modeling processes associated with direct damage to brain tissues (as, for example, in mechanical trauma), and, possibly, treatment of brain-damaged areas with using organoid transplants grown on the basis of stem cells taken from a patient.

Despite significant progress in the prevention of mechanical brain damage in terms of ensuring transport and industrial safety, disorders of nervous activity caused by the consequences of traumatic brain injuries still pose a serious threat to human health. The key to effectively eliminating the consequences of traumatic brain injury is to treat them as a chronic process with a wide range of pathophysiological disorders, one of which is an increased risk of neurodegenerative diseases. The use of organelles to establish the relationship between trauma and neurodegeneration, as well as to deeply study the degenerative processes caused by damage to the brain tissue at the molecular, cellular and tissue levels, is justified for several reasons:

1. The impossibility of reliable reproduction of inflammatory and degenerative processes associated with brain injuries in animal models, especially in rodents. In addition to differences between humans and model animals at the molecular level, interspecies differences in brain structure are a significant factor, such as different ratios of gray and white matter, the density of neurons in the cortex, and a number of other structural differences [80]. Simulation accuracy is improved when using large animals such as pigs or primates. However, in this case, the duration of the experiment and its resource consumption increase significantly;
2. The ability to fully trace the cell path, from pluripotent stem cells to nerve and glial cells, so that changes in the architecture of the nervous tissue could be observed in real-time. In most methods of working with animal models, only a certain time ‘slice’ of structures is considered, corresponding to their state at a certain point in time;
3. Creation within the model of niches with a unique microenvironment. The cell cultures used to model the long-term effects of brain injury prior to the introduction of organoid models into practice had a quite simple structure. For the most part, these are single-layer or multilayer structures containing one or more cell types [81]. Organoid models, due to their complex structure, are able to reproduce the microenvironment specific to cells in the composition of an organ, so they have greater potential for research compared to cell cultures [82,83].

Organoid models are actively used to study the influence of the microenvironment on the emergence and development of brain tumors [82,84]. Organoid models of brain tumors make it possible to consider the process of tumorigenesis in the brain as a set of interactions between microglia and other types of glial cells, stromal cells, neurons, cancer cells proper, recruited macrophages associated with tumors, as well as the influence of the blood-brain barrier and extracellular matrix components on tumors [82]. The use of a similar approach in relation to neurodegenerative diseases is justified by the fact that in both cases the process is of a chronic nature, occurs with the involvement of cells of surrounding tissues and is associated with the loss of the immune system’s ability to maintain homeostasis at the tissue and cellular levels, namely, the destruction of defective cells and stopping their impact on healthy cells [15,82,85].

One of the microenvironmental factors that are common for both brain tumorigenesis and neurodegenerative diseases is local inflammation. The key component of these reactions is RAGE receptors (Receptor for Advanced Glycation end products). In addition to that, these receptors play an important role in the development of diabetes, as well as heart and kidney failure [86]. Advanced glycation end products (AGEs) may be present in food or produced endogenously in biological systems. Their formation is associated with chronic neurodegenerative diseases such as Alzheimer’s disease, Parkinson’s disease, multiple sclerosis and amyotrophic lateral sclerosis [86,87]. The role of glycosylation end products in neurodegeneration is their ability to bind to AGE-specific receptors and the ability of their precursors to induce the so-called ‘dicarbonyl stress’, which leads to protein cross-linking and damage [88,89].

**Figure 3:**
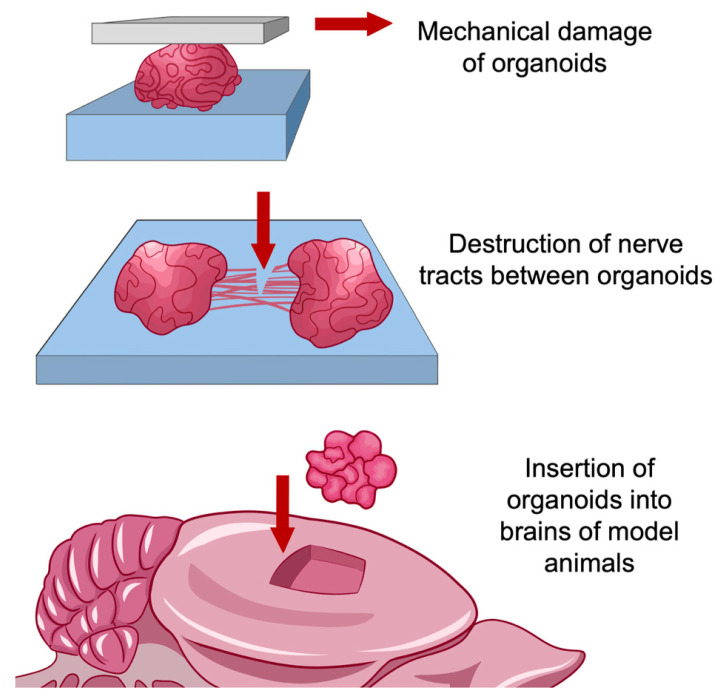
Scheme of possible use of organoids for modeling and potential treatment of traumatic brain injuries. Organelles can be used as separate systems, as interconnected units, and as part of model animal organisms.

It is highly important to note that organoid models are suitable not only for modeling the consequences of traumatic brain injuries but also for their elimination (Figure 3). Particularly a method was proposed for growing organoids corresponding to the cerebral cortex, and their further transplantation to the site of damaged areas [81].

## 8. Conclusions

The results of the data analysis on neurodegenerative disease models suggest that all types of models have characteristic properties that make them optimal tools for studying one or another aspect of neurodegenerative diseases. Bacterial and yeast models have shown themselves to be universal and unpretentious ‘protein factories’, suitable not only for studying evolutionarily conservative molecular mechanisms but also for the synthesis of transgenic proteins responsible for the onset and course of degenerative processes in the human body. The advantages of animal models (both invertebrates and vertebrates) lie, first of all, in the ability to show the effect of neurodegenerations on the body as a complex system with many aspects, including complex behavior. Organoid models are the most promising type. Their main advantage is the ability to trace the development of degenerations as sequential, chronic processes. Besides, organoid models have demonstrated their applicability not only as models but also as potential sources of transplants for the treatment of neurodegenerative diseases and conditions leading to them.

**Figure 4:**
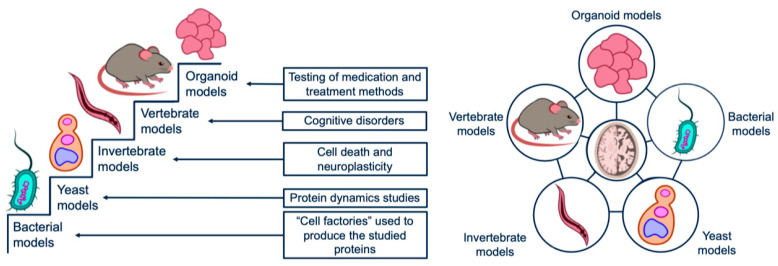
Two ways of a complex model systems organization. Left—‘step by step’, where each model for the study of a certain characteristic is a separate step, allowing to use of information from the previous step to study more and more complex processes gradually. The second way is a ‘network’. With this approach, studies are carried out with several types of models simultaneously, and experiments with each specific type are adjusted in conformity with the data obtained from other models.

At present, there is no universal model that can reflect all aspects of such a complex process as neurodegeneration. Therefore, it is advisable to use a complex consisting of models of different origins, simultaneously exploring not only processes at the molecular and cellular levels, but also the influence of environmental factors, such as the composition of the microbiome, stress and intellectual load. This complex can be organized in two forms (Figure 4).

The first form appears as a kind of ‘step-by-step’, where each model is a separate step, allowing one to use information from the previous step to gradually study more and more complex processes. Thus, transgenic bacterial models would serve to produce a protein of interest, yeast to study the dynamics of abnormal proteins, invertebrates to monitor processes such as cell death and neuroplasticity, mammals to cognitive impairment, and in vitro systems to test drugs and treatments.

**Figure 5:**
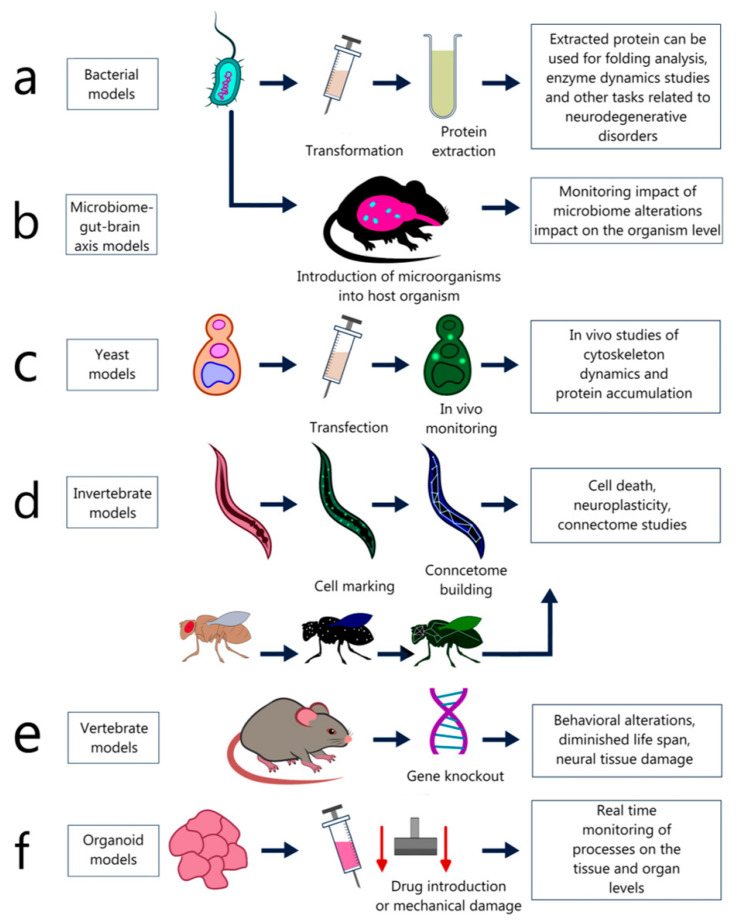
Examples of models used to study neurodegenerative diseases: (**a**) Prokaryotic organisms are used to produce various neurodegeneration associated proteins (‘protein factories’); (**b**) Prokaryotes are used to create models for studying the ‘microbiome-brain-gut’ axis, which makes it possible to determine the influence of the microbiome and the molecules produced by it on the nervous system; (**c**) Yeast (*S. cerevisiae*) is actively used to create models for studying the proteins accumulation and degradation involved in neurodegeneration; (**d**) Invertebrates (*C. elegans* and *Dr. melanogaster*) serve as a platform for analyzing the cell death, neuroplasticity and the connectomes creation; (**e**) Vertebrates allow researchers to observe the external manifestations of a particular neurodegenerative pathology in the behavior changes, life expectancy, anatomical damage to the nervous tissue of nervous system certain parts; (**f**) Organoid models make it possible to test drugs and therapeutic approaches to the neurodegenerative diseases treatment directly on human material.

The second approach, based on the “network” organization of a complex of model systems, is, in our opinion, more preferable. With the “network” approach, work is carried out simultaneously with several types of models, and work with each specific type is adjusted in accordance with data obtained from other parallel models (both more highly organized and less organized). An example is the creation of an in vitro continuous culture system that includes both human cells and microorganisms, the composition of which is adjusted in accordance with data on motor disorders that develop during life in mice with impaired intestinal microflora. A detailed analysis of the literature data presented in this review allows us to state that the ‘network’ approach to the combination of individual model systems seems to be more promising and fruitful both from the point of view of fundamental biology and diagnostics and therapy (Figure 5).

Thus, a remarkable example of the application of the ‘network’ approach to models’ organization is described in detail by Ambrosini et al. [90]. The authors point to their successful application of the method of intestinal microbiome continuous cultivation (healthy or with neurodegenerative diseases) in combination with an organoid system grown from intestinal tissues. To do this, an intestinal biopsy is performed and individual crypts are extracted from the biopsy. Then organoids (called ‘colonoids’ by the authors) are grown from these crypts, on which a miniature copy of the normal/disturbed microbiome (gut-on-a-chip) is grown, after which complex interactions are studied [91,92,93]. In addition, Shcheglovitov and Peterson in their review [94] suggest possible ways to combine two independent models: neurons or organoids derived from pluripotent stem cells (iPSCs) of patients, and Danio fish as a platform for the growth of human neurons or organoids. The authors believe that such an approach to combining models can be a breakthrough in the creation and testing of medicines. The cited works are pioneering, so the application of the “network” approach to the organization of models in the study of neurodegenerative diseases has few examples so far. Nevertheless, the ’network’ approach to modeling neurodegenerative processes will provide the most optimal and effective progress in neuropathology research, solving a number of practical problems [94].

Summarizing the data above, we can conclude that the future of neurodegenerative disease research belongs to the ‘network’ models. The ‘network’ organized complex models are the most promising both for the fundamental investigation of the whole complex of causes leading to neurodegenerative disease development and for progress in the creation and development of approaches to their prevention and treatment.

## Data Availability

Not applicable.
